# Ancient DNA Comes of Age

**DOI:** 10.1371/journal.pbio.0030056

**Published:** 2005-02-15

**Authors:** Henry Nicholls

## Abstract

Ancient DNA enables researchers to study the genetics of populations in the past; despite difficulties in its extraction, aDNA reveals that evolution is even more complex than we had imagined

The study of genetic material from ancient specimens was, in its early years, dominated by a race to sequence DNA from extinct species like the dodo and the woolly mammoth. Now that the supply of these crowd-pleasing curiosities has run dry, scientists are starting to ask new questions of ancient DNA (aDNA) that are revealing how the genetic make-up of prehistoric populations changed through time. These findings look set to trounce assumptions about how evolution really unfolded. However, there is still concern that many studies are not paying enough attention to the exacting protocols needed to overcome the technical challenges of the discipline and to defend it from the ridicule that has plagued it in the past.

In 1994, while *Jurassic Park* was still taking in millions of dollars at the box office, scientists claimed to have extracted and sequenced DNA from an 80-million-year-old dinosaur [1]. When sceptical researchers took a look at the sequence, it turned out to be of human rather than dinosaur origin. “To make that mistake, you'd have to try really, really hard,” says Alan Cooper, head of the Henry Wellcome Ancient Biomolecules Centre at Oxford University in the United Kingdom. If you think you've sequenced some dinosaur DNA, the first thing you'd do is run a phylogenetic analysis on it, he says. “Had they done that properly, with any mammal at all involved in the tree,…they would have found that their sequence was grouping with the mammals and not with the reptiles or the birds,” says Cooper. Perhaps they'd watched Michael Crichton's inventive fiction one too many times, he suggests.

## Setting the Standards

It was this kind of bungling study that highlighted the need for an exacting protocol that would steer researchers around the significant pitfalls posed by DNA decay and contamination. A list of “authenticity criteria” emerged during the 1990s, aimed at preventing similarly bogus claims from entering the literature [2]. This list includes stringent laboratory controls; cloning of products amplified by polymerase chain reaction (PCR); replication of results from a second, independent extract; and, for really new or unexpected results, replication of results by an independent research group.

Such requirements have allowed work on aDNA to move on and mature. Now, it's possible to focus on the really interesting questions that aDNA can answer. “What we're able to do with ancient DNA is really look at evolution,” says Cooper. The fossil record can only hint at how evolution unfolded. “It just shows you there's a bear and then there's not a bear,” he says. “It doesn't show you where it came from or what the relationship between the groups is.” By contrast, aDNA can do just that, giving researchers a window onto the population genetics of the past and revealing how evolution really played out. And the signs are that descriptions of the evolutionary process based on the fossil record and modernday gene pools are far too simple. “The modern data is clearly misleading us,” says Cooper. “Evolution is much, much more complex and dynamic than we would hope.”

## Thinking Big

Because of the decay that occurs with time, there is a limit to how far back aDNA can gaze (Box 1). “Your ideal preservation conditions are something that falls under ice, freezes instantly, and stays frozen until you get it,” says Cooper. “As soon as we get up to 2 million [years ago] we can't get anything to work, and that's even under deep-frozen conditions.” But within the past 60,000 years, there are several major evolutionary events that are worth studying—including a glacial maximum around 18,000 years ago, the invasion of the New World by humans about 12,000 years ago, and a global mass extinction about 11,000 years ago. These relatively recent events should be a good model for working out how similar events affected genetic diversity throughout evolutionary history.

**Box 1. Decay**

As soon as an organism dies, nucleases get the better of repair enzymes and rapidly digest strands of DNA. Under certain conditions, such as rapid desiccation, freezing, or high salt concentrations, the nucleases are inactivated before the damage is done. Even if some DNA is left intact, however, radiation, oxidation, and hydrolysis can still cause damage. These processes mean that ancient DNA specimens are like a four-letter alphabet soup, says Cooper. Very little of the original DNA remains, which is why most aDNA studies focus on mitochondrial rather than nuclear DNA: there are simply more copies of mitochondria, so the chances of getting an aDNA sequence are that much higher. Furthermore, chemical changes to the DNA fragments that remain cause additional problems: the PCR is often fooled into inserting inappropriate bases when it is copying an ancient template strand. “Amplification of DNA molecules older than one million years of age is overly optimistic,” note Pääbo and his colleagues [11].

In spite of these difficulties, several groups are hoping to work out ways to spot aDNA damage and set about repairing it. Since the 1980s, Svante Pääbo and Tomas Lindahl have made several stabs at removing glitches in aDNA using purified repair enzymes, filling in the gaps between sequences and then joining them together to resurrect something like an original sequence. “Sometimes it has seemed to help, but nothing really reproducible has come out of it,” admits Pääbo. One approach has, however, been successful at repairing DNA damage that occurs with time. Cross-links can form between reducing sugars and amino groups, he says (Figure 4). Such cross-links can sometimes be broken using the chemical N-phenacylthiazolium bromide, releasing PCR fragments that would otherwise be tied up.

The amount of repair that's possible will never be able to restore the DNA sequence of an extinct species *Jurassic Park*–style, but it should allow researchers to ask even more profound questions of aDNA. “In five years, I think we'll see some repair methods really get going,” says Cooper.

**Figure 1:**
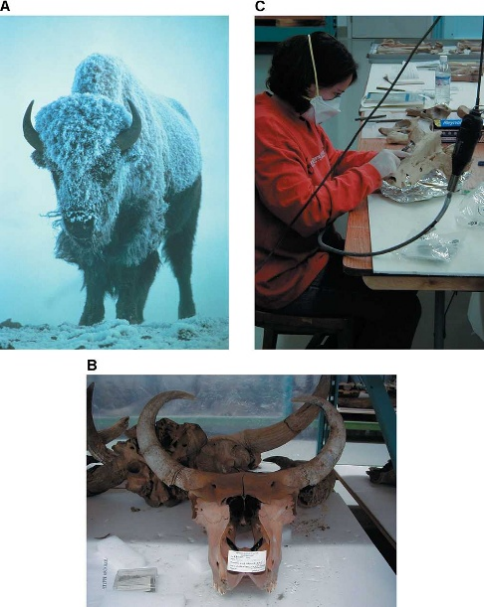
How Did Bison Really Evolve? (A) A modern bison (Bison bison), (B) the skull of an extinct bison ancestor, and (C) extraction of aDNA from a bison bone. (Images: [A] Steve Malowski, United States Fish and Wildlife Service, [B and C] Henry Wellcome Ancient Biomolecules Centre, Oxford University)

Cooper's latest work has analysed DNA from over 400 bison fossils from Beringia—the frozen wastes between eastern Siberia and the Canadian Northwest Territories [3]. “What we've done is carbon-date a shitload of bison and get DNA out of them.” It's the largest aDNA study to date, he says (Figure 1). The icy conditions mean that good quality mitochondrial DNA could be extracted from most of the specimens. The bison could also be dated accurately. This allowed Cooper and his colleagues to trace the changes in the bison genetic diversity from 150,000 years ago to the present. It was even possible to predict the effective population size throughout this period of bison evolution. “Our analyses depict a large diverse population living throughout Beringia until around 37,000 years before the present, when the population's genetic diversity began to decline dramatically,” they note.

This finding challenges some common assumptions. It has been argued that modern bison are descended from Beringian bison, but Cooper's data suggest otherwise. “All modern bison belong to a clade distinct from Beringian bison,” he and his colleagues report. Furthermore, the dramatic decline in the numbers of bison occurs long before humans arrive on the scene, scuppering the idea that hunting pressure was primarily responsible for the demise of the bison. As the glacial maximum approached 18,000 years ago, the cooler, dryer conditions were probably responsible for the downturn in the bison population, argues Cooper. “Climate change is giving the animals an absolute whacking,” he concludes.

**Figure 2:**
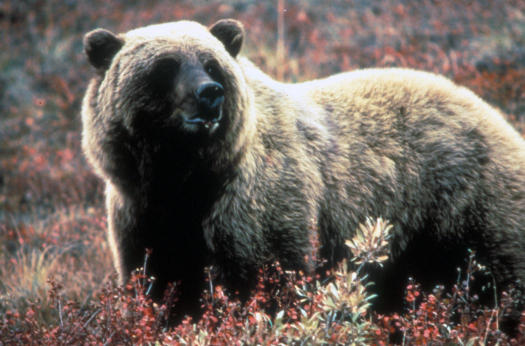
What Are the Real Evolutionary Origins of the Brown Bear (Ursus arctos)? (Image: John Nickles, United States Fish and Wildlife Service)

A similar analysis of brown bear DNA excavated from permafrost and cave deposits in the Arctic is also challenging conventional evolutionary wisdom [4]. Being able to get both a radiocarbon date and some DNA from a specimen pins a particular genetic sequence to a particular moment in time. These data suggest that genetically and geographically distinct groups of bear have replaced each other relatively often during the last 60,000 years. Regional extinctions and replacements seem to be tied to climate change and competition with the much larger short-faced bears, the authors argue (Figure 2).

Recent analysis of aDNA from Haast's eagle has also thrown up a surprising result. This New Zealand giant had a wingspan of up to three metres and a weight of around 14 kilograms, says Michael Bunce, an anthropologist at MacMaster University in Ontario, Canada. Analysis of aDNA from 2,000-year-old specimens indicates that this extinct creature is closely related to the little eagle from Australia and New Guinea, which typically weighs less than one kilogram. The common ancestor of these two eagles lived as recently as 1 million years ago, he and his colleagues estimate [5]. “It means an eagle arrived in New Zealand and increased in weight by 10–15 times over this period,” says Bunce. “Such rapid size change is unprecedented in terrestrial vertebrates.”

**Figure 3:**
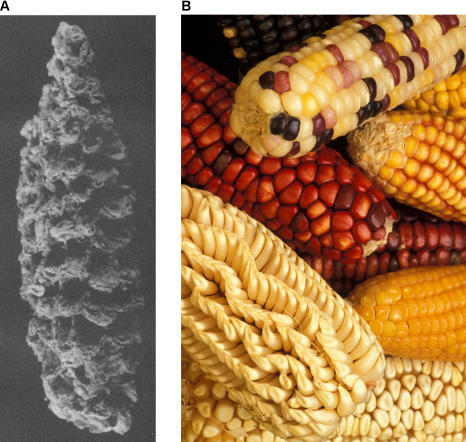
Domesticating Maize (A) Maize cob from the Ocampo Caves in Mexico dated to 3,890 years before the present. aDNA can reveal the selection of traits during early maize domestication that cannot be observed in the fossil record. (B) Examples of modern maize. (Images: [A] Svante Pääbo, Max Planck Institute, [B] Keith Weller, USDA Agriculture Research Service)

In addition to illuminating these natural events, the study of aDNA can also show changes in the frequency of key genes that occurred during the domestication of crops and animals. For example, aDNA from samples of early maize reveals when certain desirable traits appeared [6]. “It's the first study of ancient DNA that looks at phenotype,” says Svante Pääbo, an evolutionary anthropologist at the Max Planck Institute in Leipzig, Germany. “One can actually look at specific genes that early humans selected during domestication of an important crop.” Pääbo's analysis suggests that the alleles typical of contemporary maize were already present in Mexican maize 4,400 years ago, so just a couple of thousand years after its initial domestication from the wild grass teosinte (Figure 3). “Quite early on, properties were selected that were not only the structure of the plant but also the biochemistry,” he says.

aDNA is also being used to decipher human origins. Mitochondrial DNA from Neanderthals looks quite different from the mitochondrial DNA of early modern humans [7]. This lends support to the hypothesis that modern humans have a “single African origin” rather than the alternative hypothesis of “multiregional evolution”, where the ancestors of modern humans bred with Neanderthals. aDNA could also, in principle, be used to shed light on the evolutionary position of the 18,000-year-old “hobbit” recently unearthed on the Indonesian island of Flores [8]. Both Cooper and Pääbo have offered to have a go at isolating DNA from the “hominid” skeleton, but the early signs are that DNA has not survived. “The somewhat moist and tropical preservation conditions make the recovery of DNA improbable,” says Peter Brown, the paleoanthropologist at the University of New England in Armidale, Australia, who led the hobbit study. Efforts to extract DNA from other bones collected at the same site as this tiny hominid have not produced results. “We have made attempts with Stegodon molars,” he says, “but so far without success.”

## Ongoing Controversy

However, in spite of the authenticity criteria and this transition towards testing the big questions in evolutionary biology, aDNA research continues to invite controversy. In 2000, a team of United States researchers claimed to have cultured a bacterium sealed inside a 250-million-year-old salt crystal [9]. For Cooper, this is the sort of study that should require replication by an independent laboratory before publication. “When we repeated that work with the same primers, we were pulling up halobacteria from everywhere,” he says. “We took some dust from the top of the natural history museum in Oxford, extracted [DNA], used their supposedly halospecific primers and extracted a whole bunch of sequences, including some that fell within their diversity.” This strongly suggests, says Cooper, that the bacterium that was cultured was a modern bacterium, rather than an ancient specimen. “I can't see any logic for having 250 million years without any evolution.”

But Russell Vreeland, a microbiologist at West Chester University in Pennsylvania and first author of the salt-crystal study, is adamant that his methods were exacting. “The probability of having a contaminant in our sample was one chance in a billion,” he calculates. “If you use a Band-Aid today on your skin or your children, you are 1,000 times more likely to have an infection from that Band-Aid than I am to have a contaminant.” It's completely unscientific to argue that the cultured bacterium was a result of contamination simply because it resembles modern bacteria, says Vreeland. “That's throwing out the baby with the bathwater. If you can show that nothing has penetrated your sample and the DNA is inside, then the age of the DNA has to be equal to the age of that rock,” he says. “I think you can make your criteria so stringent that you miss reality.”

Others are alert to this danger. Sticking rigidly to the authenticity criteria can be a problem, argues Tom Gilbert of the Department of Ecology and Evolutionary Biology at the University of Arizona. “[The criteria] can both hinder the publication of good studies that do not adhere to all the criteria, and also enable the publication of erroneous results that adhere strictly to them,” he says. Part of the problem is that many referees of aDNA papers do not have a background working with aDNA, so are inclined to use the authenticity criteria as a checklist rather than critically evaluating each bit of research on a case-by-case basis. For example, he says, a recent high-profile study that followed all the criteria found that aDNA from two Cro-Magnon-type humans was very similar to DNA from modern humans [10]. But this could just mean that the specimens were contaminated by modern humans. “As no information was provided on the sample's handling history,” says Gilbert, “it becomes impossible for a reader to decide whether the sequences are authentic or contaminant” (Box 2). Such papers will continue to appear as long as the authenticity criteria are used by authors and referees as a checklist, he says. This does not mean the criteria should be relaxed, he adds, but they should be used in a more intelligent way.

**Box 2. Contamination**

Most tissues under the scrutiny of the aDNA researcher will contain not only DNA from the organism of interest, but also DNA from bacteria, fungi, and all sorts of other organisms. Of course, most of this confusion can be cleared up using a speciesspecific primer in the PCR to amplify DNA from just one genome. “You're after a needle in a haystack,” says Alan Cooper, “but fortunately that's what the PCR technique is so good at doing— sorting through that haystack for you.”

A lot of focus has been placed on contamination in the laboratory. This “external contamination” is a particular problem because low levels of aDNA can easily be outnumbered by just a tiny amount of high-quality modern DNA floating around the lab. One of the criteria of authenticity—separating the site of DNA extraction from the site of PCR amplification—is designed to minimise this.

In addition, there is “innate contamination” that has occurred to the sample before it even reaches the laboratory. This is much harder to control and is a real problem for those working on human origins because it's difficult to tell what is ancient human DNA and what is a modern contaminant. Bone is like sponge. “In life, bone is about 8% air. In death,…40% or 50% of it is air,” says Gilbert. So if an archaeologist digs up and washes a bone from some human ancestor, it's almost certain that modern DNA will find its way into the centre of the bone [12]. Some archaeologists have even been known to lick bones to determine their porosity, he says. “If they're licking bones, God knows what else they're doing to things.” This variable and unrecorded handling history of many museum specimens, means that contamination is difficult to rule out.

This explains why many aDNA researchers focus on unusual species like the bison or the cave bear: these animals are extinct, so the chances of contamination are nil. It's easier to come up with real results that the scientific community will accept, says Gilbert. There will always be a temptation to work on humans, however, because it's these studies that grab the headlines, he says.

But allowing authors the freedom to use the criteria as they see fit could come at a cost, says Cooper. “The trouble with a case-by-case basis is that it basically equates to no standards, because then people will do what they feel like doing and we're back to the 1990s again,” he says.

## Agreement

This ongoing disagreement over how aDNA studies should be judged does, however, stem from a common concern. As more and more biologists come to appreciate the unique ability of aDNA to probe the evolutionary process, it is more important than ever to stress the immense challenges of working with just a few fragments of degraded DNA that might have come from several different sources. It is obvious why an archaeology lab might want to set up its own aDNA facility. But this is like creating molecular biologists without a license, says Pääbo. “You wouldn't buy an accelerator and say 'I will now start doing my own carbon dating,'” he says. “You have to really have experience working with low copy number.”

However, in spite of the continual problem of eager but inexperienced biologists trying to extract DNA from specimens in the university museum, there is a sense that aDNA is starting to fill in the gaps in our understanding of key moments in evolutionary history. So at the start of 2005, as aDNA research enters its 21st year, the discipline is, perhaps, coming of age.

**Figure 4:**
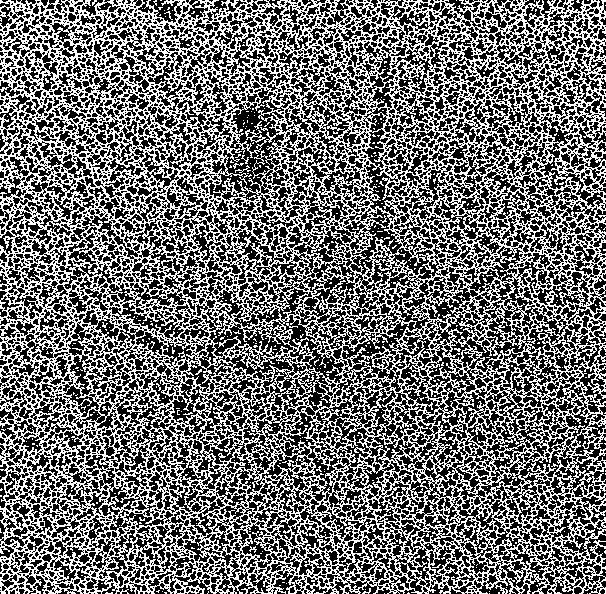
Cross-Linked DNA Extracted from 4,000-Year-Old Liver of an Ancient Egyptian Priest Called Nekht-Ankh (Image: Svante Pääbo, Max Planck Institute)

## Abbreviations

aDNA: ancient DNA
PCR: polymerase chain reaction
